# Novel *In vitro* Procedures for Rearing a Root-Feeding Pest (*Heteronychus arator*) of Grasslands

**DOI:** 10.3389/fpls.2016.01316

**Published:** 2016-08-30

**Authors:** Ivan Hiltpold, Ben D. Moore, Scott N. Johnson

**Affiliations:** Hawkesbury Institute for the Environment, Western Sydney University, PenrithNSW, Australia

**Keywords:** grasses, insect rearing, plant pests, root herbivores, soil pests

## Abstract

Optimizing plant protection against insect herbivory relies on testing plant defense mechanisms and how the insect response to these defensive strategies. Such experiments benefit from using insects generated from standardized rearing protocols since this reduces stochastic variation. Such protocols can be challenging to devise, however, especially for root herbivores. These insects generally have complex and long life cycles, which are often only poorly described. Moreover, using field-captured root herbivores is often suboptimal because it involves extensive excavation from sites selected by chance (their location is not obvious) and larval stages are frequently indistinguishable beyond the family level. We investigated *in vitro* procedures to improve the availability of the African Black Beetle (ABB) *Heteronychus arator*, an invasive alien pest in both Australia and New Zealand. Native to Africa, this scarab beetle has established in Australian and New Zealand grasslands, pastures, and crops. Adults feed on the stem of young plants just beneath the soil surface. During the mating season, gravid females lay eggs in the soil, giving rise to larvae feeding on grass roots, causing severe damage, and impairing plant growth. Here, we propose laboratory approaches to collect eggs from field-captured adult beetles, to hatch eggs, and to rear neonate larvae to adults. We propose that these methods will provide plant scientists and entomologists with a better and more controlled supply of ABB larvae for laboratory and field assays. In turn, this will assist with the collection of important information for the management of this insect pest and enhanced protection of plants in crop and grassland ecosystems.

## Introduction

Terrestrial plants can allocate up to 90% of their biomass to the production of belowground structures (root, rhizomes, and storage organs; Blossey and Hunt-Joshi, 2003). Insect herbivores, native or invasive, feeding on belowground plant organs not only affect net ecosystem primary productivity but also plant physiology, function, and growth (Brown and Gange, 1990; Blossey and Hunt-Joshi, 2003; Van Der Putten, 2003; Stein et al., 2010). Emerging evidence suggests that plants respond very differently to attacks above- and below-ground, since the nature of damage to the plant is very different (Johnson et al., 2016). Research has been hampered by the cryptic habitats of below-ground herbivores, but recent progress in various techniques such as X-ray tomography (Johnson et al., 2004), spectrometry (e.g., Rostas et al., 2015), and isotopic diet labeling (e.g., Traugott et al., 2008; Hiltpold et al., 2014) have allowed plant scientists and entomologists to improve their knowledge and understanding of root herbivory and the ecological impact of soil-dwelling insects on ecosystems (Johnson et al., 2013).

Plants suffer excessively from belowground herbivory as root damage can result in (i) a decrease in nutrient and water uptake (e.g., Riedell, 1990; Hou et al., 1997), (ii) disproportionate resource losses (Johnson et al., 2016), (iii) diversion of assimilates away from shoot growth for the re-growth of below-ground structures (e.g., Soler et al., 2012; Zvereva and Kozlov, 2012), (iv) increased susceptibility to water stress (e.g., Gange and Brown, 1989), and (v) reduced mycorrhizal association (Bennett et al., 2013) and increased infection by root pathogens (van Dam, 2009). In grasslands, primary productivity losses to root herbivory can be up to 25% (Seastedt and Murray, 2008), and this is often due to scarab beetle larvae. For instance, it is estimated that the collective biomass of soil-dwelling scarab larvae pests is equivalent to or even exceeds that of sheep in some Australian pastures (Britton, 1978).

Research into plant defense of belowground structures has been hampered by the lack of a suitable model insect root herbivore for experimental work. An ideal model organism should (i) be representative of a broader group of organisms (in this case, insect root herbivores), (ii) be amenable to experimental manipulation, and (iii) be available at a reasonable cost. The greatest obstacle in developing an insect root herbivore model in grassland ecosystems at present is availability. Reliable, standardized methods for rearing grassland root herbivores in sufficient numbers are not available. Excavation in the field is very laborious as root herbivores generally exhibit patchy distributions (Frew et al., 2016) rendering the localisation of infested sites and collection of larvae in the field laborious and time consuming. Plant protection research would greatly benefit from a large, uniform, and predictable supply of insects of all life stages throughout the year (e.g., Fisher and Bruck, 2004).

Here, we investigate procedures for capturing, maintaining and rearing the African Black beetle (ABB) *Heteronychus arator* Fabricius (Coleoptera: Scarabaeidae). ABB is a scarab considered as a major pest of grasslands, pastures, turf, and agriculture in the Southern hemisphere. Known as the Black beetle in Africa, it was accidentally introduced to Australia [first record in 1938, Matthiessen and Ridsdill-Smith (1991) and New Zealand (Todd, 1959)] where it became a major, albeit sporadic, pest in pastures (Todd, 1959; King and Watson, 1982).

In Australasia, ABB is a univoltine pest spending most of its lifespan below-ground. Adults emerge in the last months of summer (February–March) and become sexually mature in spring (September–November). During this period, ABB flies above-ground to mate and select appropriate oviposition sites. Oviposition site selection is influenced by host preference for certain grass species (i.e., *Paspalum dilatatum* Poir., *Lolium perenne* L.; King et al., 1981a). Eggs laid in the ground start hatching in late spring–early summer (November–December; Jenkins, 1965; Mercer and King, 1976; King et al., 1981c; Matthiessen and Ridsdill-Smith, 1991). Larvae feed on decaying organic matter and roots of grasses (King, 1977), rendering them more susceptible to pathogens, drought events and pulling by grazing vertebrate herbivores. ABB adults also feed on plant tissues and can cause significant damage by feeding on the bases of grass tillers (Watson and Marsden, 1982), crop plants (Jenkins, 1965), and tree seedlings (Loch and Floyd, 2001). In Australia, ABB is known to feed on over 190 cultivated grass species in 33 genera (Hangay and Zborowski, 2010), potentially making it a good model for plant scientists. In addition, it is a medium size scarab thus representative of a large proportion of insect root herbivores. Finally, Australasian populations likely arose from limited introductions, resulting in a fairly genetically uniform meta-population. More details on the ecology of this insect are discussed in Frew et al. (2016).

Probably because beetles are easier to identify, collect and are available over a longer period of time, most studies on the impact of ABB on plant biology have been conducted with adults (e.g., Sutherland and Greenfield, 1978; Russell et al., 1982; Matthiessen and Learmonth, 1998; Popay and Baltus, 2001) in various agricultural ecosystems (e.g., Matthiessen and Learmonth, 1998; Loch and Floyd, 2001). However, only few studies have looked at larval ABB behavior and its impact on plants (e.g., Sutherland and Hillier, 1974; Sutherland and Greenfield, 1978; King et al., 1981a), probably because the hidden and patchy distribution of this pest. Here, we present a set of techniques to mass collect eggs from field-trapped ABB beetles and describe comprehensive rearing methods to obtain each developmental instar of the insect, from egg to adult.

## Materials and Methods

### Field Trapping

In 2014 and 2015, two campaigns were undertaken to trap adult ABB in the field, to establish laboratory colonies. Light traps (Supplementary Figure S1, Australian Entomological Supplies Pty. Ltd., Coorabell, NSW, Australia) were placed in a pasture typical of those used for grazing in the Sydney region, located at the Hawkesbury Campus site (Western Sydney University, Hawkesbury Campus, Richmond, NSW, Australia). Each trap consisted of two sections. First, a white plastic container (230 mm height, 260 mm inner diameter), fitted with a funnel (160 mm height, 260 mm outer diameter, 30 mm funnel neck inner diameter), which was placed on the ground. Second, a vertically oriented 12 V 8 W black light fluorescent tube (Hitachi Group, Japan) attached to three clear plastic vanes (370 mm × 110 mm) cut to fit the funnel. The fluorescent tube was connected to a light-sensitive switch (Australian Entomological Supplies Pty. Ltd., Coorabell, NSW, Australia), which automatically activated the trap at dusk and deactivated it at dawn. The switch was connected to a rechargeable 12 V 7 Ah battery (CP1270EB battery, Vision Group, China).

In 2014, 2–3 traps were set up nightly from September 25th to December 12th. In 2015, insects were trapped from September 14th to December 23rd. During both campaigns, captured insects were collected every morning and transferred to the laboratory. Traps were not deployed on nights with heavy rainfall or strong wind.

### Colony Maintenance

**FIGURE 1:**
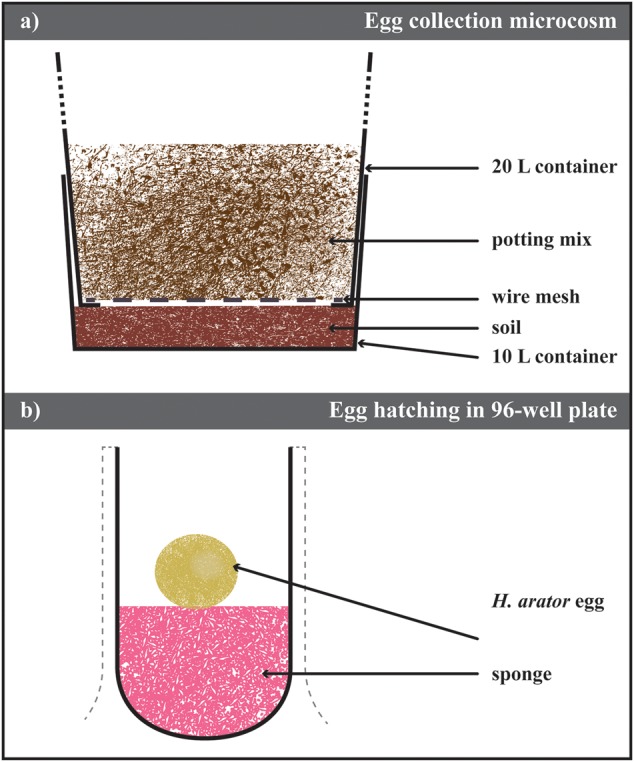
**Laboratory maintenance of *Heteronychus arator* population. (a)** Schematic drawing of the microsm used to collect eggs. It consisted of two containers inserted one into the other. The bottom of the top container was replace with a metal mesh allowing the insect move to the bottom one containing soil from the field site of the insect collection. Eggs were recovered from this layer of soil after sieving. **(b)** Schematic drawing of the methodology used to hatch eggs. Eggs were laid on a moist piece of sponge and stored until hatching. Details of both protocols in the text.

Adult ABB recovered from the field were first placed on a 500 μm sieve (Impact Test Equipment Ltd., UK), and rinsed with tap water to remove antagonists (e.g., mites, entomopathogenic nematodes, or fungal spores on the cuticle). They were then counted and released into microcosms (**Figure 1A**, Supplementary Figure S2). Each microcosm consisted of a first container (Maxi Pail 10 L Plastic Pail, 28 cm diameter, 25 cm height, Bunnings Warehouse, NSW, Australia) filled with 5 cm (King et al., 1981b) of 8:2 (w/w) autoclaved soil (Yarramundi Loam, from the site where the beetles were trapped) and water. The bottom of a second container (Maxi Pail 20 L Plastic Pail, 28 cm diameter, 41 cm height, Bunnings Warehouse, NSW, Australia) was removed in four pieces to form a 1 cm wide, 20 cm long cross, holding a wire net (5 cm × 1.0 mm mesh, Whites Group, Australia). This second container was inserted into the first one and filled with 8:2 (w/w) autoclaved potting mix (Oscmocote^®^, Scotts LLC, USA) and water. The central part of the top container lid was removed and replaced with insect mesh net (Cyclone Insect Screen, Bunnings Warehouse, NSW, Australia) to prevent beetles from escaping. Adult ABB were allowed to fly and move in the microcosm. Beetles were fed with carrots swapped every three days. Dead ABB on the surface of the potting mix were removed. Every other week, all the beetles were taken out the potting mix, sprayed with water to remove antagonists and placed back in the microcosm with new autoclaved substrate. The colonies were maintained in a greenhouse at 22°C, 65% RH, with natural photocycle.

### Beetle Oviposition and Egg Collection

Every 3 days, the soil from the bottom container of the microcosm was removed and sieved through stacked 2 mm and 500 μm-sieves (Impact Test Equipment Ltd., UK) with low-pressure water. Eggs of the ABB were retrieved with soft entomological forceps and placed in a Petri dish (10 cm diameter, Greiner Bio-One GmbH, Germany) on moist filter paper (Grade 1 Whatmann^TM^, GE Healthcare Australia, Parramatta, NSW, Australia). The Petri dish was then sealed with Parafilm^®^ (Bemis Inc., USA) and stored in the dark at 6°C until used. Every 2 weeks, dishes were checked for mold and symptomatic eggs discarded. Filter papers were maintained moist.

### Egg Hatching Protocol

Similar to the approach of Matthiessen and Learmonth (1998), eggs were hatched in 96-well plates (Greiner Bio-One GmbH, Germany; **Figure 1B**). A small piece of synthetic sponge was fitted into the bottom of the desired number of wells, up to one third of the well depth. Sponges were moistened with 3% sodium hypochlorite solution as a fungicide. The hatching plate was kept in the dark at 22°C. Eggs were monitored daily and kept moist until they hatched. Neonates were transferred to the rearing containers (details in the following section). Cumulative degree-days *dd_D_* for egg hatching were calculated as

$$\begin{array}{c} dd_{D}\;=\;(T_{D}\;-\;THR)\;+\;dd_{D-1} \end{array}$$

where *T*_D_ was the temperature of the considered day, *THR* the estimated developmental threshold (10°C, King et al., 1981d), and *dd*_D-1_ the cumulative degree-day of the previous day.

### Rearing Protocol

The first kind of rearing container consisted of a 70 ml flat-bottom specimen jar (Techno Plas Pty Ltd, Australia) filled with 8:2 (w/w) autoclaved potting mix (Oscmocote^®^, Scotts LLC, USA) and water. To avoid competition and larval cannibalism (King et al., 1981c), a maximum of two ABB neonate larvae were placed in each container. The lids of the rearing containers were perforated with four 3 mm holes to allow airflow. The containers were stored at 22°C and emptied onto a sheet of black plastic every week. The survival of the larvae was recorded and live immature insects were placed back in the containers, with new moist potting mix. Fine strips of carrot were provided on top of the potting mix (King et al., 1981d) and changed every other day.

**FIGURE 2:**
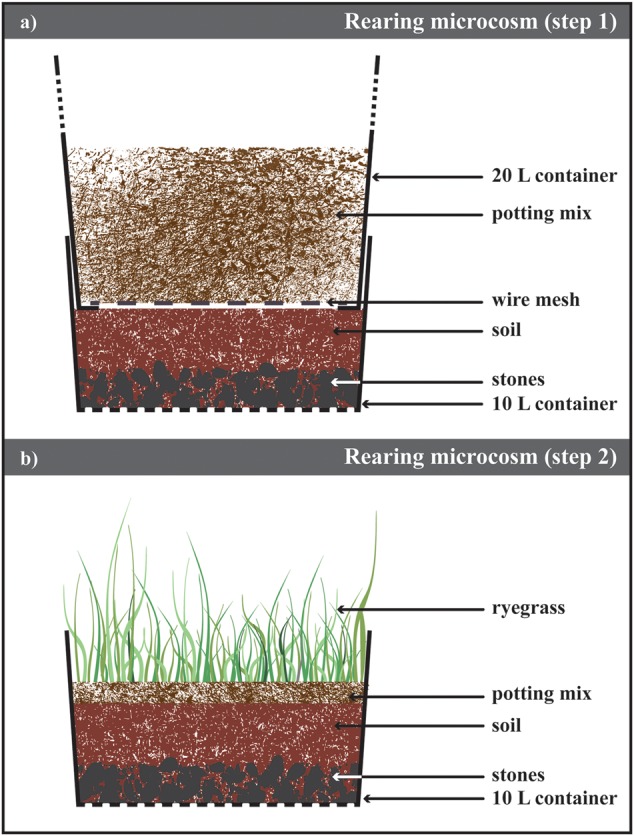
**Laboratory rearing of *Heteronychus arator*. (a)** Schematic drawing of the microsm used to rear *H. arator* from eggs to beetles. It consisted of two containers inserted one into the other. The bottom of the top container was replace with a metal mesh allowing the beetles move to the bottom one containing soil from the field site of the insect collection and stones to ensure the drainage of the excess water. **(b)** After 2 weeks, the top container was removed and ryegrass sawn on a layer of potting mix. This microcosms were stored until adult emergence.

To overcome the low larval survival rates recorded with the technique described above, an alternate, less intrusive approach was tested in 2015. Modified from the methodology to maintain the adult colony in laboratory conditions (see “Colony Maintenance”), the bottom container was adapted to hold ABB larval instars until their metamorphosis to adults (**Figure 2A**; Supplementary Figure S3). First, 3 mm diameter holes were drilled in the bottom of the container to allow water drainage. Then, a layer of about 3 cm of autoclaved gravel (2 cm < particle size < 3 cm) was covered with 15 cm of 8:2 (w/w) autoclaved soil (Yarramundi Loam, from the site of collection of the beetle) and water. At this stage, the top container (as described in section, Colony Maintenance), containing ABB adults in potting mix, was inserted above the bottom part of the microcosm and beetles were allow to lay eggs in the layer of soil.

Every 2 weeks, the top container with beetles was placed on top of a new bottom container prepared as described above. About 5 cm of autoclaved potting mix was placed in the substituted container holding newly laid eggs (**Figure 2B**; Supplementary Figure S3). In order to provide second and third instar larvae with suitable food (King, 1977; King et al., 1981a), 1 g m^-2^ of long-rotation ryegrass *Lolium multiflorum* Lam. (Poales: Poaceae; cultivar Barberia, Heritage Seeds Pty Ltd, Australia) was sown in the potting mix. This cultivar is free of fungal endophytes harmful to the insect (e.g., Popay and Baltus, 2001). The central part of the container lid was removed and replaced with insect mesh net (Cyclone Insect Screen, Bunnings Warehouse, NSW, Australia) to prevent escape of emerging beetles. The containers were stored at 22°C and regularly watered to ensure there was enough moisture for the larval development (King et al., 1981c) and plant growth; stones at the bottom ensured the drainage of excess water. The presence of emerging ABB beetles was confirmed three times a week and degree-days to emergence were recorded (details provided in section, Egg Hatching Protocol).

### Statistical Analyses

All statistical tests described below were performed in R (R Development Core Team, 2015). Plots were computed using the function *visreg* ({visreg} package).

#### Beetle Oviposition and Egg Collection

The relationship between the abundance of beetles in each container and the number of laid eggs was tested by fitting an asymptotic regression to the data (*drm* function, {drc} package). The fitting of the model was tested with a lack-of-fit test (*modelFIT* function, {drc} package).

#### Egg Hatching Protocol

The effect of storage on egg hatching success was evaluated using generalized linear models (*glm* function, {*stats*} package) with a binomial distribution. The influence of storage on degree-days required for eggs to hatch was tested with the *lm* function ({*stats*} package).

#### Rearing Protocol

Differences in % beetle emergence between containers were analyzed with a Chi-Square test (*chisq.test* function, {*stats*} package). The impact of timing of egg laying (which varied across containers) on the degree-days required for the beetles to hatch was tested with an ANOVA (*lm* function with “containertID” as factorial descriptor, {*stats*} package) to evaluate any change in egg quality over the ovipostion period.

## Results and Discussion

**FIGURE 3:**
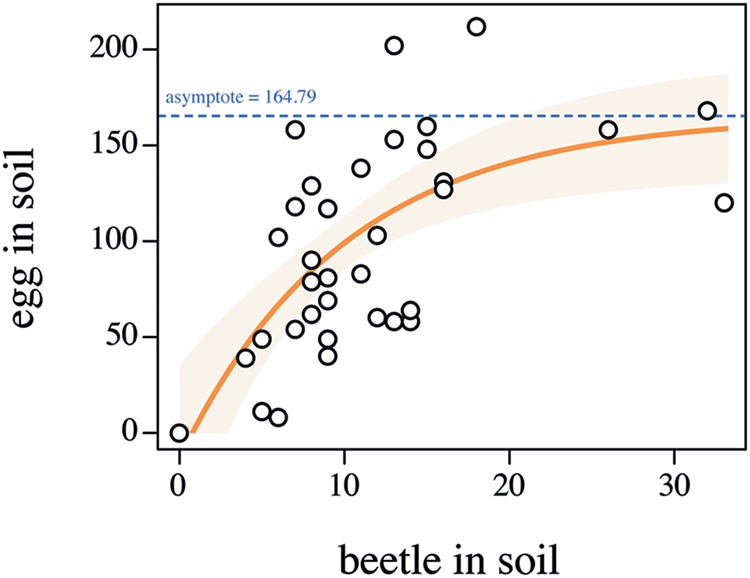
**Relationship between the number of *Heteronychus arator* beetles in the soil in the lower section of the microcosm and the number of laid eggs.** Open circles indicate the observed data. The light orange zone represents the 95% confidence interval of the fitted model (orange solid line). The dashed blue line represents the model asymptote.

In 2014, 370 beetles were captured in the field (172 males, 198 females). In the laboratory, a total of 170 beetles were observed in the soil in the bottom containers (where eggs were laid) in the egg-laying experiment. Assuming all of these were female (i.e., the most conservative estimate of fecundity), the minimum beetle fecundity was 19.98 eggs per female (3,398 recovered eggs). This is higher than fecundity levels observed in the field (ca. 12 eggs per female, Matthiessen and Ridsdill-Smith, 1991), which suggests the colony maintenance protocol used was successful. Indeed our assumption that all insects were female suggests that fecundity could well be higher than 19.98 per insect. The number of eggs found in the soil layer was positively correlated with the number of beetles present in that same layer (**Figure 3**, *F*_17-3_1 = 0.9391, *p* = 0.5416). Interestingly, the number of recovered eggs seemed to reach an asymptote around 165 eggs indicating that conspecific density might be used as a cue by ABB females to limit their oviposition, possibly to guarantee enough resources to their progeny (**Figure 3**). Such density-dependent fecundity has been observed in other beetle rearing set-ups (Peters and Barbosa, 1977) and should be accounted for while establishing an ABB laboratory colony. Preliminary attempts to collect eggs in the absence of the field soil layer failed, suggesting that ABB females require particular substrate conditions to lay eggs. This could be observed in other beetle species laying eggs in soil [e.g., Flower beetles (Coleoptera: Cetoniinae), McMonigle, 2012] and might be a required step as long as detailed knowledge on the biology and chemical ecology of the insect is not available.

**FIGURE 4:**
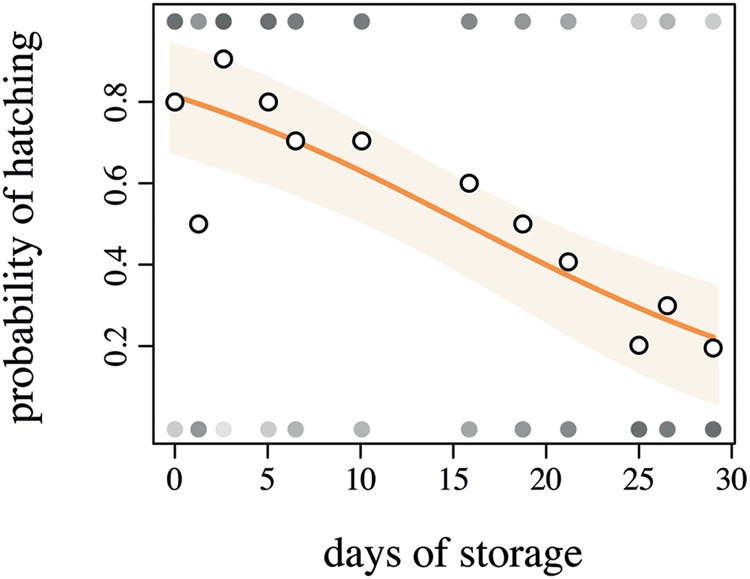
**Logistic regression between the days of storage at 6°C and the probability of *Heteronychus arator* eggs to hatch.** Open circles indicate the observed data, closed circles indicate the number of hatched or non-hatched egg (in a shade of gray where white = 0% and black = 100% hatching). The light orange zone represents the 95% confidence interval of the fitted model (orange solid line).

**FIGURE 5:**
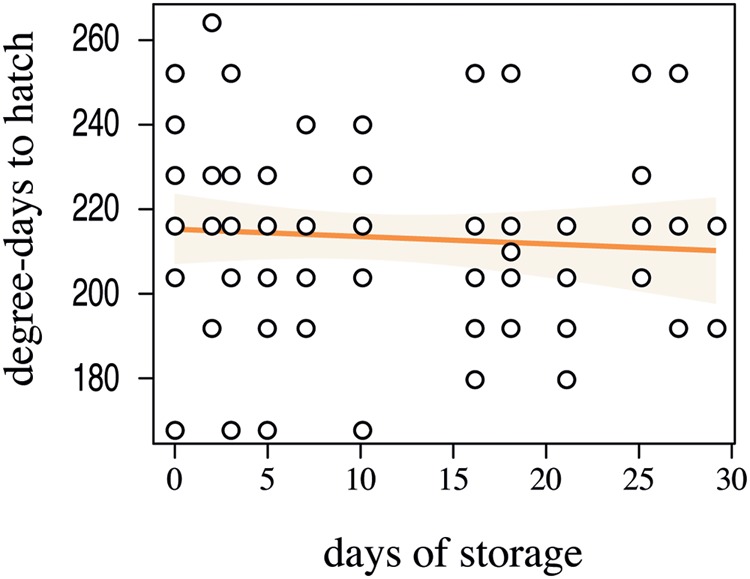
**Linear regression between the days of storage at 6°C and the degree-days required for *Heteronychus arator* eggs to hatch after removal from storage.** Open circles indicate the observed data. The light orange zone represents the 95% confidence interval of the fitted model (orange solid line).

In the 96-well plates, 46% of the eggs hatched. Storage time of the eggs, even in a cold environment, significantly impacted the hatching probability (**Figure 4**, *Z*_1-129_ = -4626, *p* < 0.001, *R*^2^ = 0.23). Given the lower hatching probability, we would advise that the eggs should not be stored longer than 15 days at 6°C in order to guarantee higher hatching rates. The cumulative degree-days to hatch was slightly negatively correlated with the number of days in storage, yet this correlation was not significant (**Figure 5**, *F*_1-66_ = 0.3349, *p* = 0.5648). The negative slope of the model (–0.1759) suggests that ABB egg still very slowly developed at 10°C, yet this temperature seems to be an appropriate estimate of the developmental threshold of ABB. Storing ABB eggs at 10°C could extend the period of viability of eggs, as compared to storage at 6°C (**Figure 4**), and prolong shelf-life of the eggs. Storage in the fridge was originally tested in the hope of being able to delay hatching to ensure the availability of insects over an extended period of time, as it is done with some other insect species, especially with biological control agents [e.g., Trichogrammatidae species (Hymenoptera), Spínola-Filho et al., 2014]. As this approach was unsuccessful, it should be considered whether varying the temperature at which the beetle colonies are maintained in the laboratory can potentially delay the oviposition, but this has yet to be tested.

Despite a relatively good egg-hatching rate, the survival of the neonate ABB larvae in the specimen jars vas very low (ca. 5%, 4 larvae out of 68 hatched eggs). King et al. (1981d) demonstrated that younger ABB larval instars mainly feed on ubiquitous decaying organic matter. We hypothesize that the organic matter provided to the hatched ABB was too coarse or too fresh to be suitable for consumption by the larvae, resulting in a very low survival. An alternative explanation could be injuries caused by handling. Entomological forceps were used to manipulate ABB larvae and, despite their softness, the tweezers might have injured some larvae during transfer and handling, adding to the natural mortality.

In 2015, a total of 90 beetles (52 males, 38 females) were captured from the field site. Based on the fecundity calculated in the laboratory set-up in 2014, an estimated total of 759 eggs were laid. In total, 148 adults emerged and were collected from the four containers. No differences in the number of emerged ABB adults were observed between containers (χ^2^ = 3.521, *df* = 3, *p* = 0.318). The observed survival in this laboratory set-up (19.5%) was similar to what has been measured in the field (Matthiessen and Ridsdill-Smith, 1991; Matthiessen and Learmonth, 1998; Matthiessen, 1999). This rate is ca. twofold higher than the estimated survival required to maintain a population to the next generation (9.6%, Matthiessen, 1999). No differences in the degree-days for ABB to emerge were recorded between the containers (*F*_1-3_ = 0.586, *p* = 0.625). The average cumulative degree-days for the adults to emerge was 1076.51 ± 8.04 SEM. This second approach offers a laboratory source of each ABB larval instars and adults. Varying the storage temperature would likely allow researchers to either accelerate or slow down the development of ABB, according to the experimental requirements (Bhuiyan and Nishigaki, 1995).

## Conclusion

Because of their biology, it is difficult to collect soil-dwelling insect pests from the field in sufficient quantities for experimentation, and until now this has limited the use of a model insect root herbivore in studies of below-ground plant–insect interactions. For many years, we and others have spent many laborious hours digging larvae or trapping adults to conduct limited experiments to unravel root-feeding insect biology. The procedures we have described here allow ABB to be reared from eggs to adults in a laboratory setting, setting this species up to be used as a model insect root herbivore. While our procedures do not improve the survival of the insect above that observed in the field, these procedures save considerable time in the field and ensure the development of larvae under uniform conditions. Controlling temperature might allow some degree of manipulation of the insect development and therefore the availability of particular desired developmental stages (i.e., larval instars) over a longer period of time. Attempts to culture ABB under semi-artificial conditions were not successful and the current method still relays on natural rearing components instead of artificial diets or medium. Despite this current weakness, this rearing approach is easy to set up, cost effective and probably applicable to other root-feeding scarabs or coleopteran insects with soil-dwelling larvae (McMonigle, 2012). Adapting existing artificial diets used in the rearing of other scarab beetle larvae, such as *Popillia japonica* (Coleptera: Scarabaeidae; Klein and Allsopp, 1994), could help in the establishment of artificial conditions. Yet the development of insect artificial diet is a laborious task, which intrinsically requires a reliable source of insect.

By making available a model root herbivore, with a generalist diet, we hope to facilitate comparative studies of root defense and herbivory across diverse plant species and environments. This in turn will enable researchers to address a neglected but vitally important aspect of plant ecology, particularly in the context of changing agricultural and land management practices, and climate change.

## Author Contributions

IH, SJ, and BM conceptualized the study. IH designed the experimental set-ups, collected beetles in the field, and conducted the laboratory experiments. IH analyzed and interpreted the data and drafted the manuscript. All authors were involved in reviewing, revision, and final approval of the manuscript.

## Conflict of Interest Statement

The authors declare that the research was conducted in the absence of any commercial or financial relationships that could be construed as a potential conflict of interest.
