# Supplementary material for: Novel In vitro Procedures for Rearing a Root-Feeding Pest (Heteronychus arator) of Grasslands
Source: Front Plant Sci. 2016 Aug 30;7:1316. doi: 10.3389/fpls.2016.01316 (PMC5003920; doi:10.3389/fpls.2016.01316)
Supplement: Supplementary file 1 [file Data_Sheet_1.PDF]

Supplementary Material

Novel *in vitro* procedures for rearing a root-feeding pest (*Heteronychus arator*) of grasslands

Ivan Hiltpold*, Ben D. Moore, Scott N. Johnson

*** Correspondence:** Ivan Hiltpold: i.hiltpold@westernsydney.edu.au

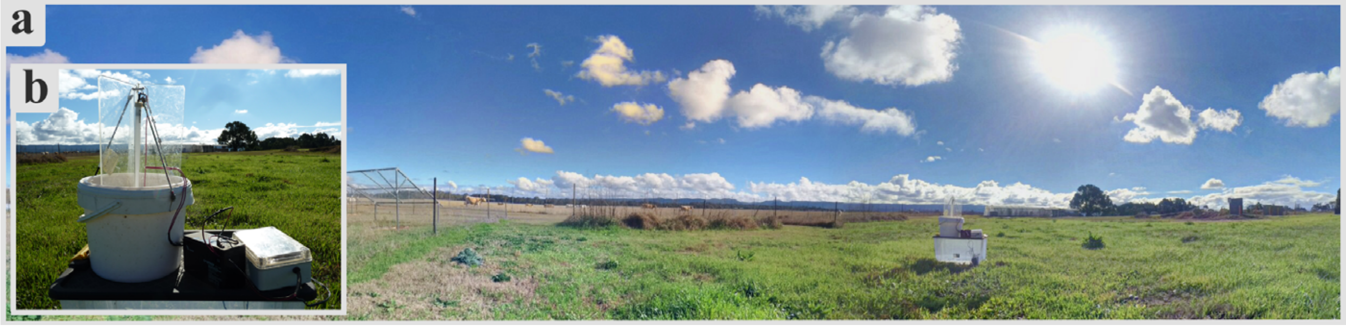

Supplemental Figure S1. a) Pasture where the traps were set up at dusk to capture *Heteronychus arator* beetles in the field at Western Sydney University, Richmond Campus experimental field facilities (full 360° picture available here, facility credits: Hawkesbury Institute for the Environment, Western Sydney University, picture credits: I. Hiltpold). b) Close-up of the light trap. Trapped adults were transferred to the laboratory to establish *in vitro* cultures of this grassland root pest. Details and dimensions in the text.

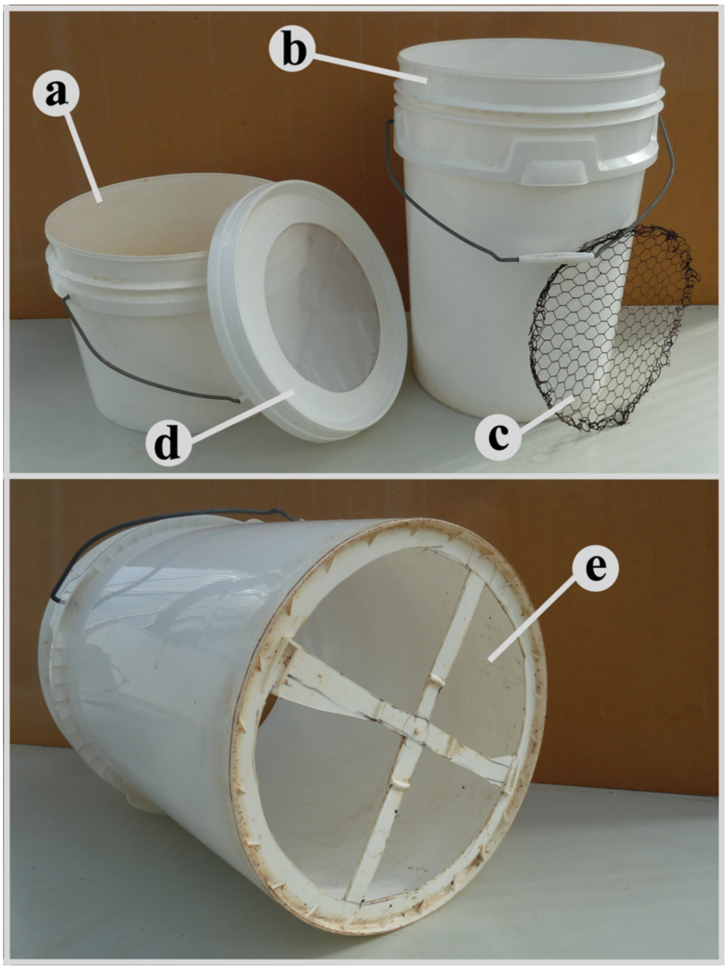

Supplemental Figure S2. Pictures of the components used to build the microcosms where eggs of *Heteronychus arator* were collected. a) Soil from the site of capture was placed in the bottom container for egg laying. b) The top container contained potting mix and *H. arator* beetles. The potting mix was held in the upper container with c) a wire net. d) The central part of the top container lid was removed and replaced with insect mesh net. e) The bottom of the top container was removed to allow beetles to crawl in the soil layer. The remaining plastic cross held the wire mesh. Details and dimensions in the text.

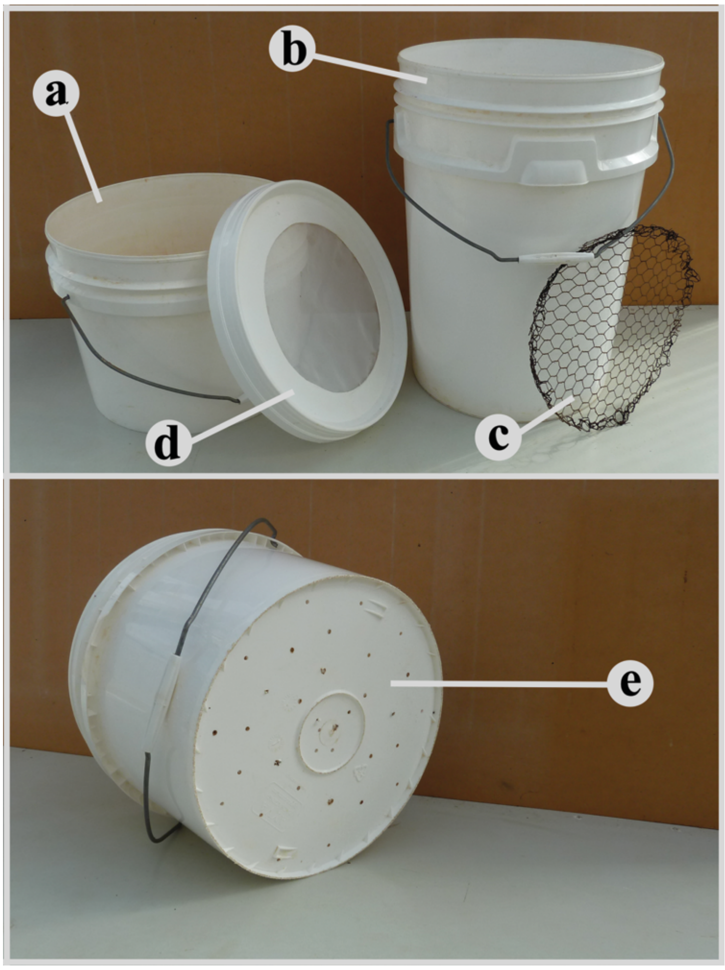

Supplemental Figure S3. Pictures of the components used to build the microcosms where *Heteronychus arator* were reared. a) Soil from the site of capture was placed on top of a layer of autoclaved stones in the bottom container for egg laying. b) The top container contained potting mix and *H. arator* beetles. The potting mix was held in the upper container with c) a wire net laid inside the bottom of the container (see Fig. S2e). Every two weeks, the top container was placed on a new bottom container. Potting mix was placed on top of the soil in the substituted container and sowed with *Lolium multiflorum* to ensure a sustainable source of food for the developing larvae. d) The central part of the top container lid was removed and replaced with insect mesh net. e) The bottom container was drilled with 3 mm holes to ensure drainage. Details and dimensions in the text.
